# ERP mismatch response to phonological and temporal regularities in speech

**DOI:** 10.1038/s41598-020-66824-x

**Published:** 2020-06-18

**Authors:** Alexandra K. Emmendorfer, Joao M. Correia, Bernadette M. Jansma, Sonja A. Kotz, Milene Bonte

**Affiliations:** 1 Department of Cognitive Neuroscience, Faculty of Psychology and Neuroscience, Maastricht University, Maastricht, The Netherlands; 2 Maastricht Brain Imaging Center, Faculty of Psychology and Neuroscience, Maastricht University, Maastricht, The Netherlands; 3 Department of Neuropsychology and Psychopharmacology, Faculty of Psychology and Neuroscience, Maastricht University, Maastricht, The Netherlands; 4 Centre for Biomedical Research (CBMR)/Department of Psychology, Universidade do Algarve, Faro, Portugal

**Keywords:** Language, Cognitive neuroscience, Psychology

## Abstract

Predictions of our sensory environment facilitate perception across domains. During speech perception, formal and temporal predictions may be made for phonotactic probability and syllable stress patterns, respectively, contributing to the efficient processing of speech input. The current experiment employed a passive EEG oddball paradigm to probe the neurophysiological processes underlying temporal and formal predictions simultaneously. The component of interest, the mismatch negativity (MMN), is considered a marker for experience-dependent change detection, where its timing and amplitude are indicative of the perceptual system’s sensitivity to presented stimuli. We hypothesized that more predictable stimuli (i.e. high phonotactic probability and first syllable stress) would facilitate change detection, indexed by shorter peak latencies or greater peak amplitudes of the MMN. This hypothesis was confirmed for phonotactic probability: high phonotactic probability deviants elicited an earlier MMN than low phonotactic probability deviants. We do not observe a significant modulation of the MMN to variations in syllable stress. Our findings confirm that speech perception is shaped by formal and temporal predictability. This paradigm may be useful to investigate the contribution of implicit processing of statistical regularities during (a)typical language development.

## Introduction

In order to effectively deploy resources for efficient processing of incoming sensations from our environment, our brain formulates online predictions of upcoming sensory events^1–3^. This is possible through our knowledge about regularities in the sensory environment, allowing us to anticipate the consequences of an action, adapt behaviour to an upcoming event, or ease sensory processing under noisy conditions. These predictions may be formal (‘what’) or temporal (‘when’) in nature^4^. A formal prediction constitutes a prediction of the formal structure or content of an upcoming event. In speech processing, formal predictions can occur at multiple levels, such as the semantic category of a word in a sentence, or the sequence of speech sounds (phonemes) within words, the phonotactic structure. Temporal predictions on the other hand are related to the anticipation of temporally regular events. Within language, the vocalic nucleus of a syllable is often considered the perceptual beat^5^, and the metre (alternation between strong and weak beats) can be described as the alternation between strong and weak (or stressed and unstressed) syllables. Therefore, the anticipation of syllable stress may constitute a form of temporal prediction.

Formal and temporal predictions are thought to operate via distinct neural oscillatory mechanisms^4^ and structural circuits^6,7^. While the processing of formal and temporal structure of the speech signal has traditionally been studied in isolation, these features vary simultaneously in natural speech. A few studies have found variation in metre to influence semantic^8^ and syntactic^9^ processing. Furthermore, difficulties in processing the temporal structure of speech have been suggested to underlie phonological processing deficits observed in dyslexia^10–12^. It is therefore of interest to study how formal and temporal predictability may interactively influence speech perception.

Both forms of predictions are established through experience in development and may play a vital role in successful skill learning. Evidence of sensitivity to regularities of the formal and temporal structure of language can already be found in infants. Newborns within 5 days of birth are already sensitive to differences in the rhythmic structure of speech^13^, while sensitivity to statistical regularities between neighbouring speech sounds has been demonstrated as early as 8 months of age^14^. Sensitivity to syllable stress and phonotactic probability provide a crucial foundation for early language development, allowing infants to segment words in the continuous speech signal^15–17^.

Phonotactic probability continues to influence performance on a number of primarily sublexical language processes throughout the lifespan. Children and adolescents show better performance in speed and accuracy for high compared to low phonotactic probability items in nonword repetition tasks^18–20^. This effect is reversed for word learning^21,22^. Similar patterns are observed in adults, with a high-probability advantages shown for spoken nonword recognition^23,24^, nonword repetition^25,26^ and serial nonword recall^27^, but a disadvantage for high probability items in word learning^28^. This contrast between nonword repetition and word learning in both children and adults is hypothesized to be due to low probability sequences of speech sounds being more easily identified as novel words that need to be learned, effectively triggering the learning process more readily^21,22,28^.

Although these and other behavioural effects of phonotactic probability are relatively well documented (see review by Auer and Luce^29^), the role of lexical stress in speech perception, beyond guiding speech segmentation, is less well studied. When a language permits different lexical stress patterns, these may guide the resolution of lexical conflict in spoken word recognition^30^. Performance on nonword repetition has also been shown to improve for more “typical” stress patterns within the language^31^.

We aim to probe neural correlates of these processes in normally reading adults by means of a passive oddball paradigm, which is particularly suited for the investigation of experience-dependent neurophysiological changes. In a classical passive oddball paradigm, a sequence of auditory stimuli is presented to the participant, consisting of a frequently occurring standard stimulus, and an infrequent deviant or ‘oddball’ stimulus. The participant is instructed to ignore the stimuli and typically reads a book or watches a silent film to remain awake and relaxed. In this type of passive paradigm, the ERP component of interest is the mismatch negativity (MMN), a negative deflection in voltage surrounding frontocentral and central electrodes in the window 100–250 ms after the onset of the stimulus deviation^32^. While early MMN studies have used simple stimuli such as pure tones^33^, the component has also been employed to study linguistic processing, ranging from simple vowel discrimination^34^ to higher-order processes such as syntax^35^. The MMN has been interpreted as a marker for experience-dependent change detection and its timing and amplitude are indicative of the perceptual system’s sensitivity to the presented stimuli.

Oddball experiments in adults and children have demonstrated that the MMN component can be modulated by variations in phonotactic probability, i.e. the probability of the co-occurrence of phonemes in a language, where deviants with higher probability have been shown to elicit larger mismatch responses compared to deviants with low probability^36–38^. The paradigm has also been applied to study processing of syllable stress patterns in both real and pseudowords^39–42^. In languages with a strict syllable stress pattern such as Hungarian and Finnish, where stress is always on the first syllable in bisyllabic words, deviant stimuli using an illegal stress pattern elicit two consecutive MMNs, where the first is thought to reflect the missing stress on the first syllable, while the second reflects the detection of the unexpected stress on the second syllable^39,40,42^. Although variations of formal and temporal predictability simultaneously occur in natural speech, ERP studies have so far typically investigated these in isolation.

In the current study, we employed a multi-feature oddball paradigm simultaneously manipulating both formal and temporal predictions in Dutch pseudowords, in the form of phonotactic probability and syllable stress pattern, respectively. We examined the effect of violations of these predictions on the MMN response, where we expected the timing and magnitude of this response to vary with the formal and temporal predictability of the stimuli. If more predictable formal and temporal features of the stimuli (i.e. high phonotactic probability and first syllable stress) are processed more efficiently, this should lead to easier change detection, indexed by greater MMN peak amplitudes and/or shorter latencies. This has been shown for phonotactic probability^36–38^. However previous studies investigating MMN sensitivity to syllable stress have been primarily conducted in languages with fixed-stress patterns^39–42^. Those conducted in languages with variable stress patterns (e.g. English, German), primarily focussed on MMN sensitivity to specific acoustic features of syllable stress, comparing responses between naturally spoken first syllable stress standards to deviants where pitch, intensity or vowel duration is manipulated to generate second syllable stress^43,44^, or simply note the presence of an MMN to both first and second syllable stress deviants without directly comparing the two^45^. Additionally, to extend upon previous studies which investigated these features in isolation, we aimed to test whether their simultaneous manipulation would yield similar or different patterns of MMN modulations, and whether stimulus features would interactively modulate the mismatch response (i.e. whether variations in syllable stress modulate formal deviant processing and vice versa).

In summary, the current study aims to test the following hypotheses: (1) deviants differing from standards in terms of phonotactic probability (hereafter formal deviants) or syllable stress (hereafter temporal deviants) elicit an MMN, indicated by a greater negativity in response to deviants compared to standards; (2) this MMN to formal or temporal deviants is modulated by phonotactic probability or syllable stress, respectively, which may present as a larger MMN amplitude^36–38^, or shorter MMN latency for more predictable (high phonotactic probability/first syllable stress) deviants. (3) variations in predictability in the other domain (*syllable stress* for formal deviants, *phonotactic probability* for temporal deviants) may further modulate this MMN sensitivity. The analyses compared identical stimuli presented in different conditions (standard versus formal or temporal deviant), which allowed us to generalize the results beyond mere acoustic differences between the stimuli.

## Methods

### Participants

29 native Dutch-speaking participants with normal reading skills participated in the experiment after giving their informed consent. 5 participants were excluded from further analysis (1 for technical issues during recording, 1 for excessive noise in EEG data (>20% trials rejected from amplitude criterion), 2 for exclusion criteria revealed during or after participation (1 left-handed, 1 learning disability), 1 for failure to complete both study visits), leaving a final sample of 24 right-handed participants (mean age = 22.6; range = 18–30, 10 males). The study was approved by the Ethics Committee of the Faculty of Psychology and Neuroscience at Maastricht University performed in accordance with the approved guidelines and the Declaration of Helsinki.

### Stimuli

#### Pseudowords

**Figure 1:**
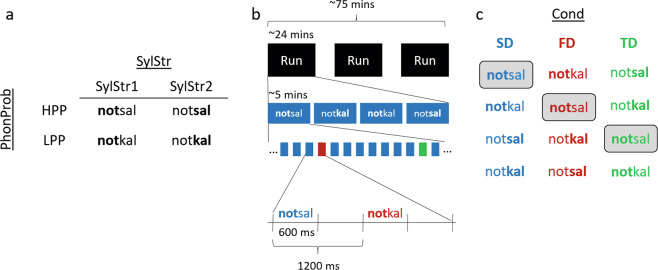
Experimental design. (**a**) Pseudoword stimuli varying in phonotactic probability (PhonProb) and syllable stress (SylStr). Bold font indicates stressed syllable (SylStr1 = first syllable, SylStr2 = second syllable. The phoneme combination ‘-ts-‘ constitutes high phonotactic probability (HPP) and ‘-tk-‘ low phonotactic probability (LPP). (**b**) Overview of experimental session. (**c**) Each stimulus is presented as standard (SD), formal deviant (FD), and temporal deviant (TD) in separate conditions (Cond), allowing the comparison of identical stimuli across conditions (example highlighted for **not**sal).

The stimuli used in the oddball paradigm were adapted from a previous paradigm employing Dutch pseudowords *notsel* and *notkel*^36,37^. These stimuli were initially constructed by calculating phonotactic probabilities using the CELEX database^46^, where the phonotactic structure ‘-*ts-’* was found to have a higher probability than ‘-*tk-’*. This stimulus pair can therefore be used to test the role of formal predictions. To add the dimension of temporal prediction to these stimuli, we additionally varied the syllable stress pattern placing the stress on either the first or second syllable, creating a stimulus quadruplet (Fig. 1a). We adapted the pseudoword pairs from notsel-notkel to notsal-notkal in order to avoid possible changes of the vowel ‘schwa’ due to stress variation. Both phonotactic constructions and syllable stress patterns are legal in Dutch but occur at different frequencies. The relative frequencies of these features are indicated in Table 1, as determined by the word frequencies of bisyllabic Dutch words containing the target phoneme structure or syllable stress pattern, retrieved from the CELEX database^46^.

**Table 1:** INL frequencies of target phoneme structures and syllable stress patterns.

	PhonProb			SylStr		
	-ts- Freq	-tk- Freq	-ts-/-tk- Ratio	SylStr1 Freq	SylStr2 Freq	SylStr1/SylStr2 Ratio
All BiSyl	94061	7787	12.09	7633058	1926345	3.962
CVCCVC	1227	0	—	292794	18817	15.560

*Note*. Frequencies represent the sum of INL (Instituut voor de Nederlandse Taal) frequencies of all Dutch words containing the indicated consonant cluster or stress pattern, for all bisyllabic Dutch words (All BiSyl) or limited to those with a CVCCVC structure, while ratios represent the ratio of these frequencies for high probability (HPP, SylStr1) compared to low probability (LPP, SylStr2) conditions. (C = consonant, V = vowel, SylStr1 = 1st syllable stress, SylStr2 = 2nd syllable stress).

#### Recording and editing stimuli

The stimuli were spoken by a female native Dutch speaker and recorded in a sound attenuated chamber using GoldWave Digital Audio Editor (sampling rate 44100 Hz, 16 bit; GoldWave Inc., St. John’s, NL Canada). Due to the scarcity of second syllable stress in bisyllabic words with a CVCCVC syllable structure (occurring only in 6% of CVCCVC words as indicated in Table 1), natural pronunciation of the pseudowords with second syllable stress can be challenging to Dutch speakers. To circumvent this issue, the speaker was instructed to pronounce the syllables of interest within the context of existing bisyllabic Dutch words, which contained the same (spoken) consonant cluster and stress pattern as the target pseudowords.

The speaker first pronounced the existing Dutch word several times to familiarize herself with it. The first or second syllable of the word was then replaced by the target syllable in the pseudoword, and the speaker was instructed to pronounce the new word with the same stress pattern as the original word. Thus, the speaker first pronounced the real word /**bad**zout/, followed by the pseudowords /**not**zout/ and /**bad**sal/ (bold font denotes syllable stress, underline denotes target syllable) to create our first syllable stress pseudoword /**not**sal/. The other syllables were constructed in a similar way: /ont**slag***/* -> /not**slag**/ & /ont**sal**/ -> /not**sal**; /**geld**kas/ -> /**not**kas/ & /**geld**kal/-> /**not**kal/ and /goed**koop***/* -> /not**koop**/ & /goed**kal**/ -> /not**kal**/. (Note that in Dutch, a syllable final /d/ is indistinguishable from a syllable final /t/ due to final-obstruent devoicing, and the /z/ in /badzout/ is pronounced as /s/). The target syllables were later spliced from these recordings and combined to create the pseudowords using Praat^47^. The matching consonant cluster at the syllable boundary ensured identical co-articulatory cues, facilitating cross-splicing of syllables to create the final pseudowords. To construct the pseudowords, excised target syllables were paired to ensure equivalent changes in pitch and intensity for both first or second syllable stress. *Notsal* stimuli were created by combining /no/ of the first syllable with /tsal/ from the second syllable to minimize acoustic artefacts within the consonant cluster /ts/ from the splicing procedures. Because of the voice-onset time preceding the /k/ in /-kal/, this was not necessary for *notkal* stimuli. The constructed stimuli were then edited to equalize for loudness (rms amplitude) and duration (600 ms). Three versions of each stimulus were created from distinct utterances of each syllable (i.e. each syllable in the final pseudowords was unique). This allowed the generalization to the target features of phonotactic probability and syllable stress beyond small acoustic variations.

### Data acquisition

#### Oddball paradigm

A passive oddball paradigm was used, where each stimulus served as the standard in separate conditions (Fig. 1b,c). Each condition contained a temporal and a formal deviant, which differed from the standard in terms of either the syllable stress or phonotactic probability, respectively. Each condition contained a total of 1,620 trials (1,332 standards and 144 deviants, or 8.9%, per deviant type). The experiment took place over two sessions. Each session consisted of three runs of approx. 24 mins, split into four blocks (one per condition) of 270 trials. Participants were encouraged to take breaks as needed in between blocks and runs. Within a block, trials were presented with trial duration of 1,200 ms (i.e. inter-stimulus-interval 600 ms). The stimuli were presented in pseudorandom order, with deviants separated by 1–8 standards. The order of blocks within each run was randomized for each participant.

#### EEG recording

EEG was recorded with BrainVision Recorder (Brain Products, Munich, Germany) using a 63-channel recording setup. Ag/AgCl sintered electrodes were mounted in an EasyCap electrode cap (EASYCAP GmbH, Herrsching, Germany) according to the 10% equidistant system, including 57 scalp electrodes, left and right mastoids for offline re-referencing, and four EOG electrodes to facilitate removal of artefacts caused by eye movements. The skin at electrode sites was prepared with NuPrep Skin Prep Gel (DO Weaver and Co., USA) and an electrolyte gel was used to keep impedances below 10kΩ. Data were recorded at a sampling rate of 1000 Hz, using Fpz as an online reference and AFz as ground. During recording, participants were seated on a comfortable chair in an acoustically and electrically shielded room and instructed to watch a silent nature documentary while ignoring the auditory stimuli.

### Analysis

#### Preprocessing

Preprocessing was performed using MATLAB 2017a and the EEG analysis toolbox Letswave 6 (https://github.com/NOCIONS/letswave6). Data were first filtered (band pass 0.5–70 Hz, notch filter 48–52 Hz) and down-sampled to 250 Hz. Noise from eye-movements, muscle artefacts, and noisy electrodes was removed using independent component analysis^48^ (ICA) with the runica algorithm implemented in Letswave 6, decomposing the signal into 63 components. The time course recorded during the breaks between blocks was removed from the data to reduce noise prior to the ICA. Artefactual components were selected for removal based on the time course and topography. A median of 19 components (~30%) was rejected per dataset (SD = 6). From the resulting data, −100 to 1000 ms epochs relative to the onset of the stimulus were extracted. After DC removal and baseline correction to the pre-stimulus interval (−100 to 0 ms), an automatic artefact rejection algorithm was applied with an amplitude criterion of 75µV over scalp electrodes to remove trials with remaining artefacts, and the data was re-referenced to the average mastoids. Deviants occurring after only one standard were excluded from analysis. Standards immediately preceding deviants were selected for analysis, resulting in up to 126 trials for each deviant type, and 252 standards for each stimulus. To allow comparing the same standard trials to both formal and temporal deviants, while ensuring equal number of trials across conditions, a random subset of standards was selected to equal the smallest number of deviants across conditions per participant. Within participants, the number of trials was equalized across conditions, leading to a final number of 99–124 trials per condition per participant.

#### ERP analysis

Trials were averaged after time-locking to the onset of the auditory deviation, corresponding to stimulus onset for temporal deviants, and the /t/-onset for formal deviants. Difference waves were calculated (deviant – standard of identical stimuli, where the standard was always time-locked to the same moment as the respective deviant). Individual and grand average difference waveforms per condition where examined to determine the time window for peak extraction (100–300 ms after /t/-onset for formal deviants, 200–350 ms after stimulus onset for temporal deviants). MMN peak latency to formal and temporal deviants was defined based on the difference waves per participant at FCz. This electrode was selected due to the well-documented frontocentral topography of the MMN^32^. Amplitude measures of the MMN were determined around the FCz peak latency for all other electrodes to ensure the comparison of the same underlying process across the scalp^49^. First an automatic algorithm in the Letswave toolbox was used to find a negative peak at FCz in the pre-specified time window. The waveforms and topography were then visually inspected to confirm the selection, or to adjust it to a more fitting peak that reflected the typical frontocentral MMN topography, within a final time window of 80–300 ms for formal deviants, and 120–370 ms for temporal deviants. Mean amplitudes (+/− 24 ms surrounding the peak) were calculated for standard and deviant waveforms at all other electrodes at this latency. Frontocentral (Fz, F1, F2, F3, F4, FCz, FC1, FC2, FC3, FC4) and centroparietal (Cz, C1, C2, C3, C4, CPz, CP1, CP2, CP3, CP4) regions of interest for the amplitude measures were selected based on the frontocentral topography of the elicited mismatch response and comparisons with previous literature^36,40,42^.

Statistical analyses were performed in R^50^ (version 3.6.3) with the Rstatix package^51^. For formal and temporal deviants individually, the MMN mean amplitudes were analysed with a 2 × 2 × 2 × 2 repeated measures ANOVA with *phonotactic probability* (PhonProb: HPP vs. LPP), *syllable stress* (SylStr: SylStr1 vs. SylStr2), *condition* (Standard vs. Deviant) and *region-of-interest* (ROI: frontocentral vs. centroparietal) as within-subjects factors. We set out to test the following hypotheses: (1) formal and temporal deviants elicit an MMN, indicated by main effect of *condition* on MMN mean amplitude, with greater negativity for deviants compared to standards; (2) this MMN is sensitive to the predictability of the stimulus features (*phonotactic probability* x *condition* for formal deviants, *syllable stress* x *condition* for temporal deviants), with more predictable features (HPP or SylStr1) showing a greater mismatch response; (3) variations in predictability in the other domain (*syllable stress* for formal deviants, *phonotactic probability* for temporal deviants) may further modulate this MMN sensitivity (*phonotactic probability* x *syllable stress* x *condition)*.

The MMN latency for both deviant types was further analyzed in a 2 × 2 repeated measures ANOVAs with PhonProb and SylStr as within-subjects factors (because the peak latency was determined based on the difference wave MMN peak at a single electrode, Cond and ROI were not included as factors in this analysis). Here we tested our hypotheses (2) the MMN latency is sensitive to the predictability of stimulus features, indicated by main effect of *phonotactic probability* for formal deviants and *syllable stress* for temporal deviants, with more predictable features (HPP or SylStr1) showing an earlier mismatch response; and (3) variations in predictability in the other domain (*syllable stress* for formal deviants, *phonotactic probability* for temporal deviants) may further modulate this MMN sensitivity (*phonotactic probability* x *syllable stress*).

## Results

**Figure 2:**
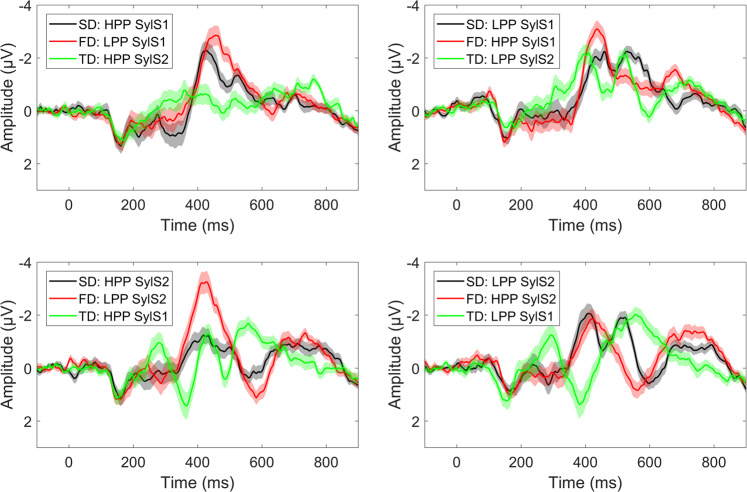
Grand average waveforms +/− SEM at a frontocentral ROI within conditions: Each panel represents one standard (black) and the formal (red) and temporal (green) deviants that were presented within a block: SD = Standard, FD = Formal deviant, TD = Temporal deviant.

Figure 2 provides an overview of the waveforms elicited by the stimuli presented within each condition (e.g. standard (SD) = **not**sal, formal deviant (FD) = **not**kal, temporal deviant (TD) = not**sal**), time-locked to the onset of the stimulus. Visual inspection of the waveforms revealed that all pseudoword contrasts elicited a mismatch response between 100–350 ms after the onset of auditory stimulus deviation. Formal deviants appeared to show a negative deflection compared to the standard in the window 100–300 ms after the auditory deviation at the /t/-onset (~350–550 ms after stimulus onset), in all conditions but LPP SylStr2 (bottom right panel), while temporal deviants showed a similar negative deflection around 200–350 ms after stimulus onset. In order to focus on MMN modulations at a more abstract level of representation, in our further analyses we compared the activity elicited by the same stimuli as standards and deviants across blocks; e.g. *not*sal formal deviant minus *not*sal standard. The following results are presented for formal and temporal deviants separately, time-locked to the auditory deviation in the respective contrasts. Amplitude analyses were performed on mean amplitudes (+/− 24 ms surrounding peak). Here we present results of the tests of our a priori hypotheses, corrected for multiple comparisons using Bonferroni-Holm correction^52^. All other effects tested in the ANOVAs (significant and non-significant), can be found in the supplementary materials.

### Formal deviants

**Figure 3:**
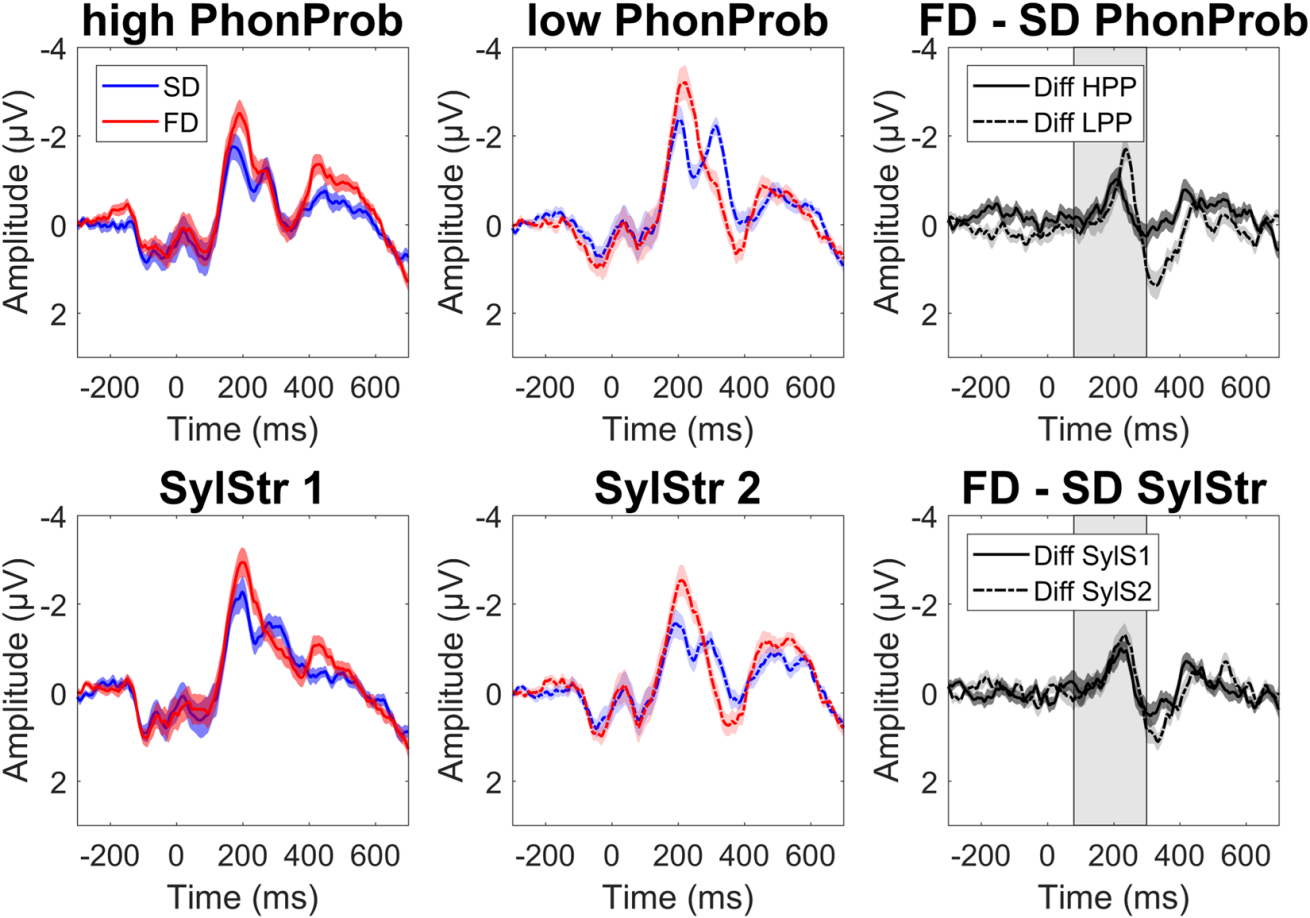
Formal deviants. Grand average waveforms + /− SEM at frontocentral ROI, time-locked to /t/-onset. Top panel is averaged across syllable stress: high phonotactic probability (red: deviant, blue: standard), low phonotactic probability, and the difference waves (dark grey: high PhonProb, light grey: low PhonProb). The bottom panel is averaged across phonotactic probability: first syllable stress, second syllable stress, and difference waves (dark grey: SylStr1, light grey: SylStr2). Shaded area in difference waves represents window for MMN peak extraction.

**Figure 4:**
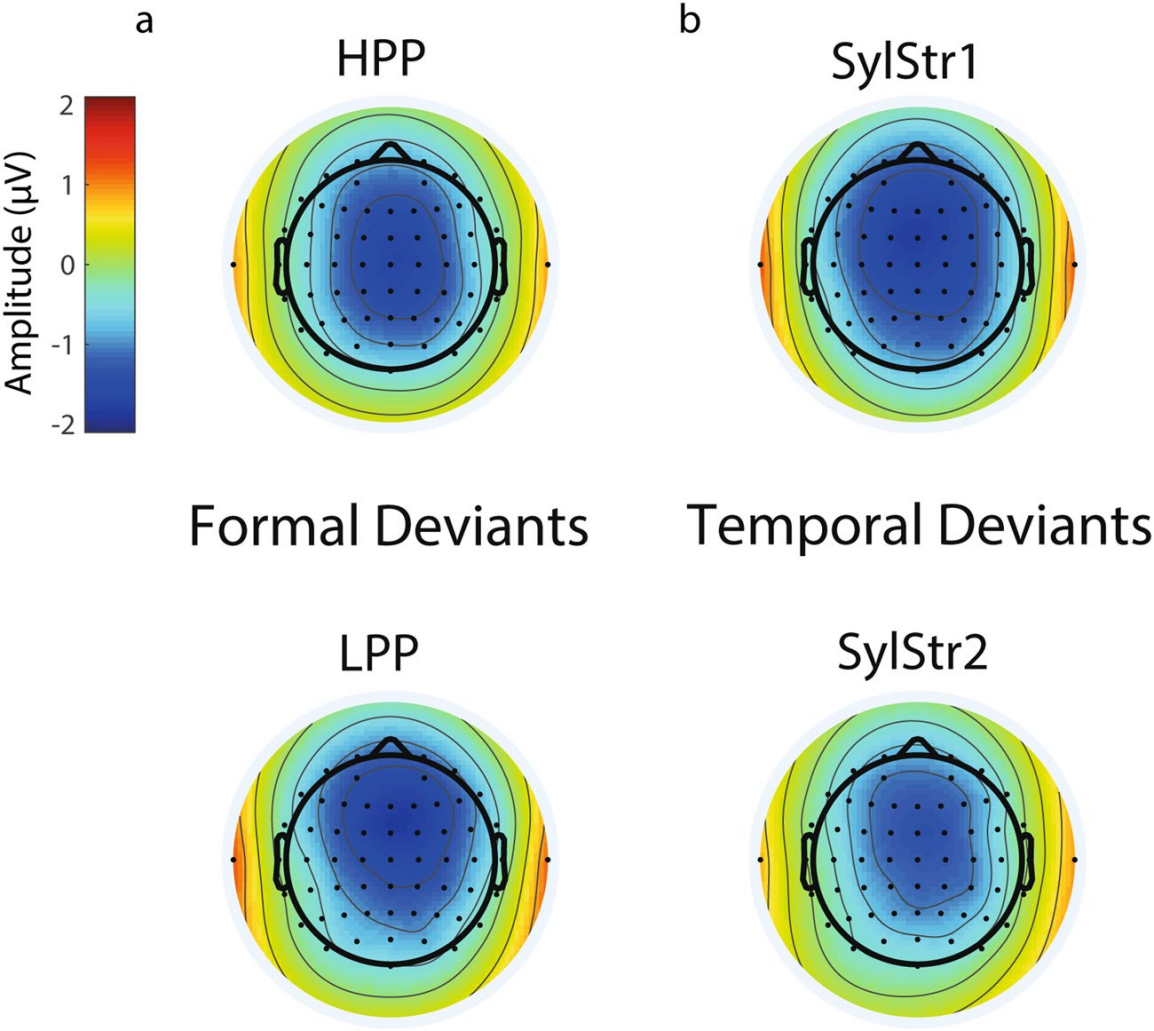
MMN topographies. Average MMN topographies of mean amplitude (+/− 24 ms) surrounding individual MMN peaks. (**a**) Formal deviants averaged across syllable stress. (**b**) Temporal deviants, averaged across phonotactic probability.

Grand average ERPs for standards and formal deviants, time-locked to the onset of the /t/ at the frontocentral ROI, are shown in Fig. 3. Both HPP and LPP formal deviants, averaged across syllable stress, showed a more negative peak response compared to the identical stimulus presented as a standard in a window around 100–300 ms after the /t/-onset, with comparable topographies (Fig. 4a). Visual inspection of the difference waves suggests an effect of phonotactic probability, with a larger mismatch response for LPP formal deviants and an earlier mismatch response for HPP deviants (top panel), but no effect of syllable stress on formal deviants (bottom panel).

**Figure 5:**
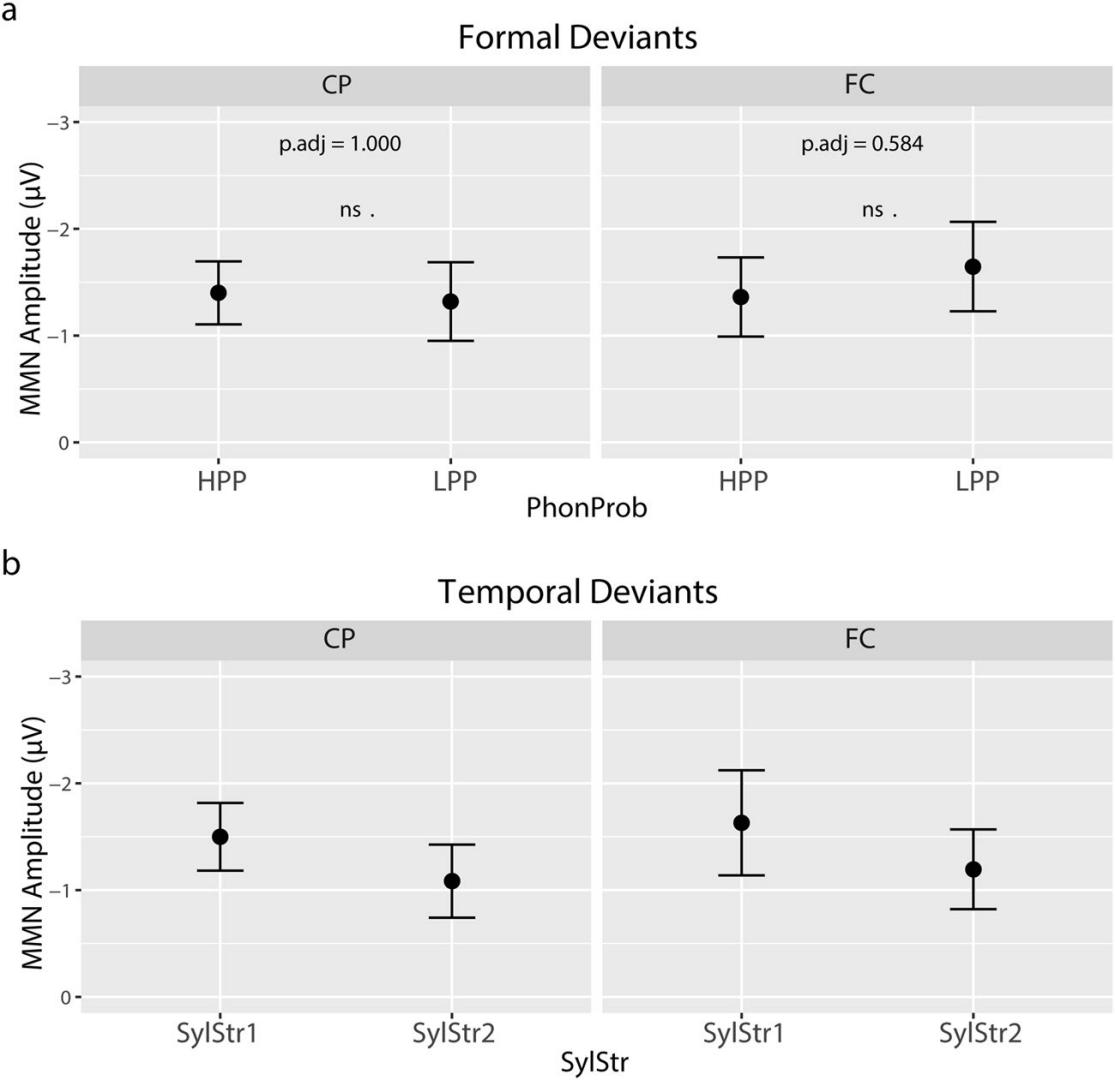
MMN mean amplitudes (Deviant – Standard). (**a**) Formal deviants, averaged across syllable stress at centroparietal (CP) and frontocentral (FC) ROIs. No significant effect of phonotactic probability on MMN mean amplitude in either ROI (posthoc two-sided paired samples t-test, Bonferroni correction). (**b**) Temporal deviants, averaged across phonotactic probability at CP and FC. No statistical comparison was made due to non-significant SylStr x Cond interaction. Errorbars represent 95% confidence interval of the mean.

The amplitude observations were statistically tested using a 2 × 2 × 2 × 2 repeated measures ANOVA (PhonProb × SylStr × ROI × Cond) on MMN mean amplitudes. The analysis of mean amplitudes revealed a significant main effect of Cond [F(1,23) = 107.642, p_adj_ < 0.001], with deviants eliciting a significant MMN, confirming our first hypothesis. The PhonProb x Cond interaction was not significant [F(1,23) = 0.187, p_adj_ = 1.000], however, a possible trend towards a three-way PhonProb × Cond × ROI interaction [F(1,23) = 8.568, p_adj_ = 0.112] is observed (Fig. 5a). Post-hoc two-sided paired-samples t-tests on the mean amplitude difference (FD – SD) comparing high and low phonotactic probability, averaged across syllable stress, did not reveal any significant effect in either the frontocentral [t(23) = 1.08, p_adj_ = 0.584] or centroparietal ROIs [t = −0.373, p_adj_ = 1.000] (while we hypothesized a larger MMN for HPP deviants, which would warrant a one-sided test, our data as seen in Fig. 3 suggest a larger MMN for LPP deviants, therefore we selected the two-sided test).

**Figure 6:**
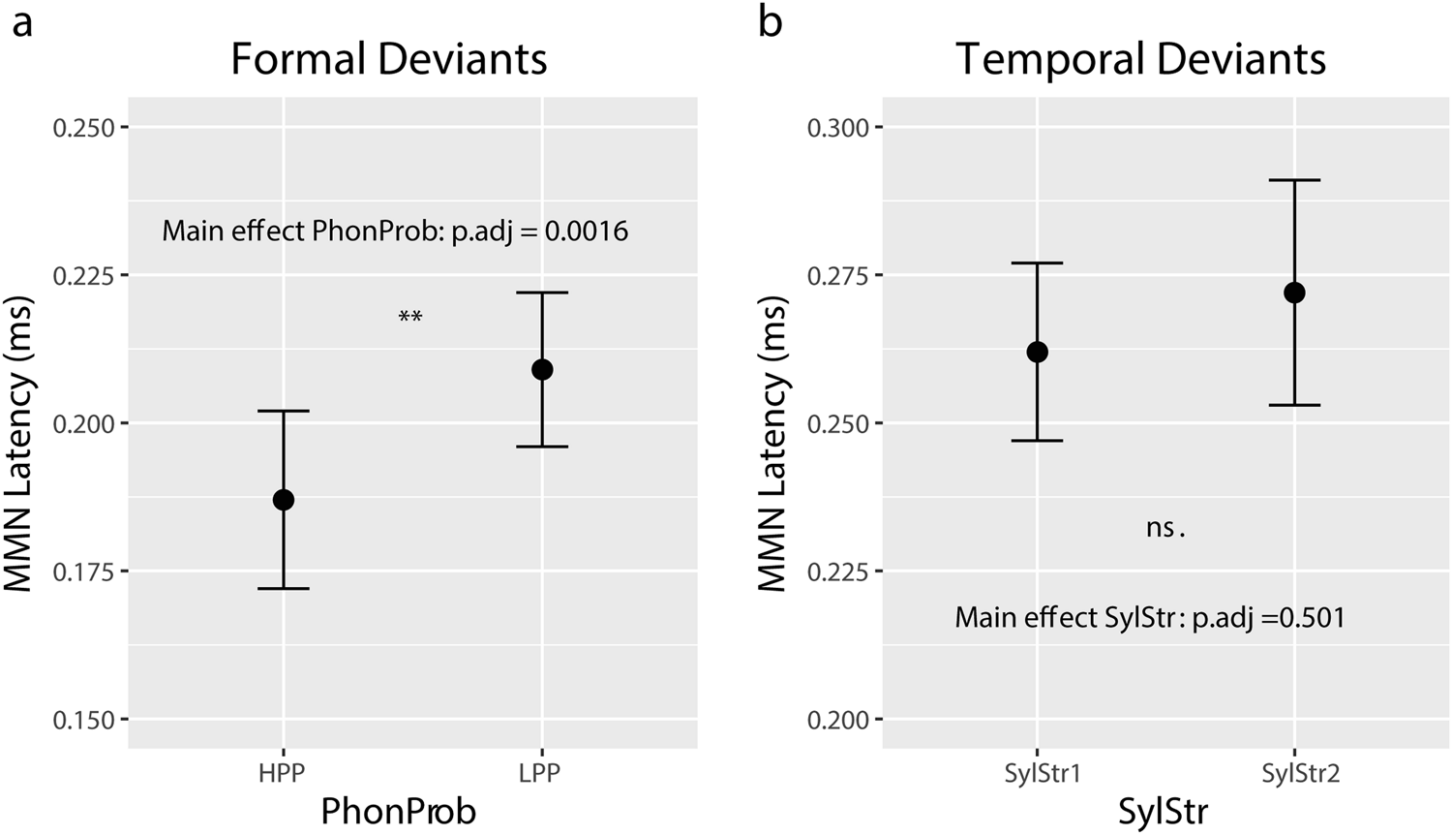
MMN latency measures. (**a**) Formal deviants averaged across syllable stress. Significant main effect of phonotactic probability (2 × 2 ANOVA, Bonferroni-Holm correction), with HPP deviants eliciting and earlier MMN compared to LPP deviants. (**b**) Temporal deviants averaged across phonotactic probability. No significant main effect of syllable stress (2 × 2 ANOVA, Bonferroni-Holm correction).

Latency observations were tested in a 2 × 2 repeated-measures ANOVA (PhonProb × SylStr) on MMN peak latency. This analysis revealed a main effect of PhonProb [F(1,23) = 16.249, p_adj_ = 0.0016], where HPP deviants show a shorter peak latency than LPP deviants (Fig. 6a). Thus, we are able to confirm our second hypothesis that the MMN is sensitive to phonotactic probability, however unlike previous studies^36–38^ which demonstrated an effect in MMN amplitude, we observe this effect in MMN latency. Neither amplitude nor latency measures show support for our hypothesis that formal deviant processing may be modulated by syllable stress. No other significant effects were observed for amplitude (Supplementary Table S3) or latency (Supplementary Table S4).

### Temporal deviants

**Figure 7:**
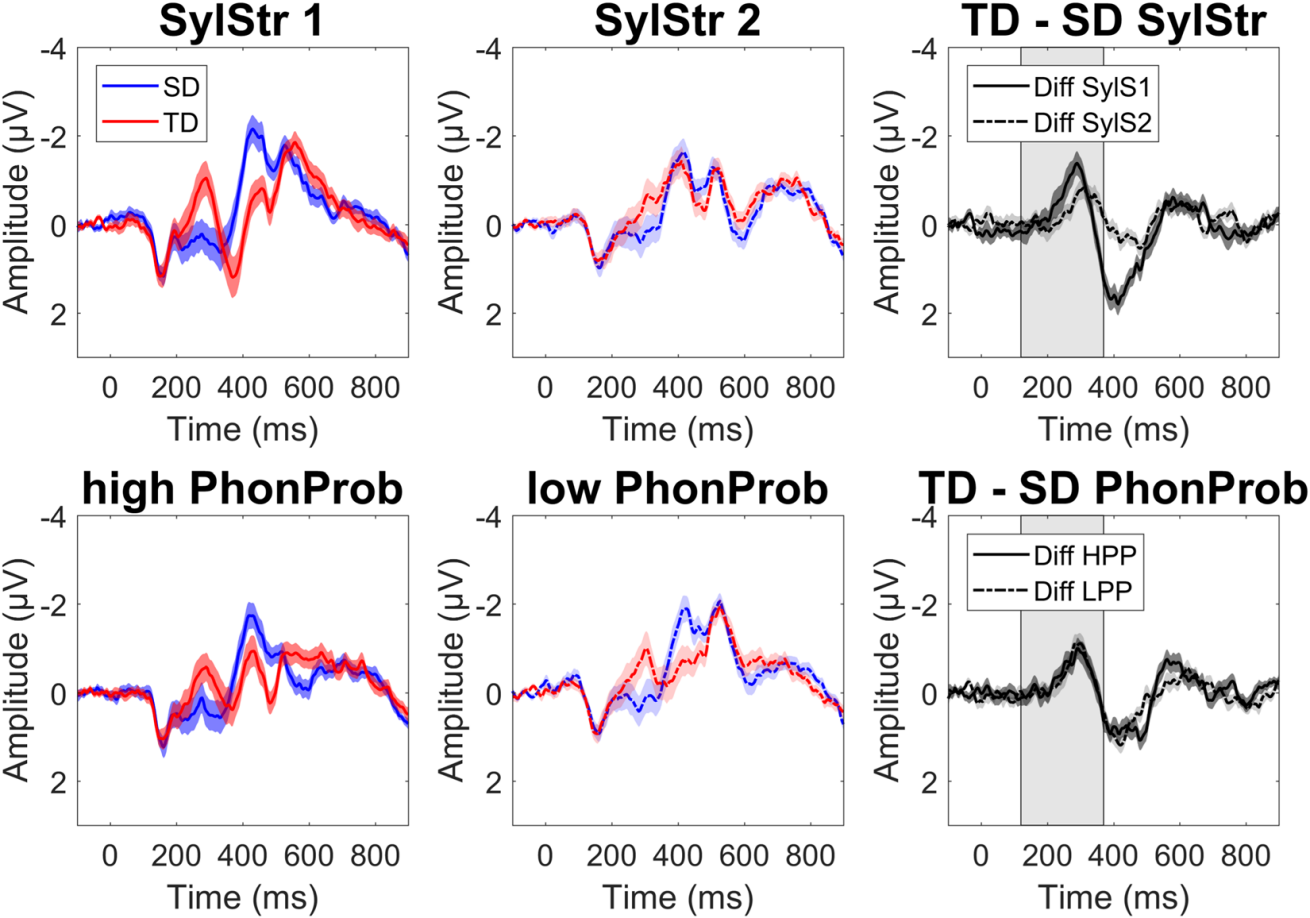
Temporal deviants. Grand average waveforms +/− SEM at frontocentral ROI, time-locked to word onset. Top panel: averaged across phonotactic probability: first syllable stress (red = deviant, blue = standard), second syllable stress, and difference waves (dark grey = SylStr1, light grey = SylStr2). Bottom panel: averaged across syllable stress: high phonotactic probability, low phonotactic probability, and difference waves (dark grey = HPP, light grey = LPP). Shaded area in difference waves represents window for MMN peak extraction.

Grand average ERPs for standards and temporal deviants time-locked to the onset of the stimulus at the frontocentral ROI are shown in Fig. 7. First and second syllable stress temporal deviants, averaged across phonotactic probability, showed a more negative peak response compared to the identical stimulus presented as a standard in a window around 200–300 ms after stimulus onset, with comparable topographies (Fig. 4b). Visual inspection of the difference waves suggests an effect of syllable stress, with a larger mismatch response for temporal deviants with first syllable stress (top panel), but no apparent effect of phonotactic probability (bottom panel).

These observations were again tested statistically with a 2 × 2 × 2 ×2 repeated-measures ANOVA (PhonProb × SylStr × ROI × Cond) on MMN mean amplitudes. The analysis of mean amplitudes revealed a significant main effect of Cond [F(1,23) = 78.159, p_adj_ < 0.001], confirming our hypothesis that the temporal deviants elicit an MMN. The SylStr x Cond interaction did not reach significance after Bonferroni-Holm correction [F(1,23) = 5.360, p_adj_ = 0.420] (Fig. 5b). No other amplitude measures were significant (Supplementary Table S7). The 2 × 2 ANOVA (PhonProb × SylStr) on MMN peak latency at FCz revealed no significant main effects or interactions (Fig. 6b, Supplementary Table S8).

## Discussion

The aim of the current experiment was to develop and test an EEG paradigm to provide a measure for formal and temporal predictions in speech perception. This was achieved by means of a passive auditory oddball paradigm, with stimuli consisting of Dutch pseudowords varying in their phonotactic probability (formal prediction) and syllable stress pattern (temporal prediction). The component of interest, the mismatch negativity, is a marker of auditory change detection modulated by experience: It is sensitive to higher-level regularities in the speech signal which are acquired during development, including phonotactic probability^36^ and syllable stress^40^. While both features have been studied in oddball paradigms in isolation, these features vary simultaneously in natural speech. We therefore aimed to examine the effect of manipulating them simultaneously, to examine whether this would yield similar or different effects on the MMN.

Based on previous ERP experiments manipulating phonotactic probability^36–38^, we predicted a larger peak amplitude in the MMN response to formal deviants with high phonotactic probability, compared to their low probability counterparts. Our current results however, do not show a significant effect of phonotactic probability on MMN peak amplitude. Instead, we observed an effect of phonotactic probability on MMN peak latency, with HPP deviants eliciting an earlier MMN than LPP deviants. A shorter peak latency, indicative of facilitated processing, has been found for other ERP components and paradigms used to investigate the neural correlates of phonotactic probability^48–50^. Faster neural processing may thus reflect a possible mechanism underlying the previously reported facilitative behavioural effect of high phonotactic probability on speech processing^23,25,26^. Therefore, although our current results do not show the same pattern reported in previous MMN studies^36^, they may be interpreted in the same context, with the shorter peak latency for MMNs to HPP formal deviants suggesting a facilitated change detection. This effect may reflect Hebbian associative learning, where more frequently co-occurring speech sounds have established more stable auditory cortical memory traces, which can then be accessed more readily^36^.

Our study differs from previous passive oddball experiments manipulating phonotactic probability in the Dutch language, which used only first syllable stress in their stimuli. This is the most frequent and natural stress pattern, occurring in around 80% of bisyllabic Dutch words, and close to 95% when including only monomorphemic words (i.e. excluding words with unstressed prefixes; determined by query in CELEX database^46^). By including the manipulation of syllable stress in our design, we hoped to examine the interaction between these two factors, and see whether manipulating them simultaneously would yield similar or different patterns of MMN responses. While we did not find any significant interaction between these factors, we can observe a trend for a larger latency effect in stimuli with second syllable stress. It is furthermore possible that the simultaneous variation of phonotactic probability and syllable stress within a condition interfered with the processes underlying the findings reported in previous oddball studies^36–38^, which may explain the discrepancy in our findings.

For the temporal domain, we predicted variations in the probability of stress patterns to follow a similar pattern to the phonotactic probability, where stress patterns with high probability (i.e. first syllable stress) would elicit a stronger mismatch response compared to the low probability variation (i.e. second syllable stress). While visual inspection of the ERPs (Fig. 6) suggests that this is the case, this comparison did not reach significance after correcting for multiple comparisons (p_adj_ = 0.420, p_uncorr_ = 0.030). Previous studies showing an effect of syllable stress on the MMN were performed primarily in languages where second syllable stress is not a legal construction in bisyllabic words, such as Hungarian^39–41^ and Finnish^42^. Here, the ‘illegal’ second syllable stress deviant is reported to elicit a double MMN response, where the first negative peak is thought to reflect the missing stress on the first syllable, and the second negative peak the response to the unexpected stress on the second syllable^39,40,42^.

While second syllable stress is rare in Dutch, it is nevertheless a legal construction in bisyllabic words, which suggests that native Dutch speakers would also process deviations in stress patterns in a different manner than speakers of Hungarian or Finnish. A study conducted in German, which also allows variability in syllable stress patterns similar to Dutch, demonstrated that both first and second syllable stress deviants elicited an MMN in adults, while infants only showed a significant MMN in response to first syllable stress^45^. While the study did not statistically compare the MMN amplitudes for different stress patterns to each other, the results suggest that regularities in syllable stress influence speech perception more heavily early in development, where it is exploited to segment the continuous speech signal into words^17^. Once we have acquired the language successfully, this feature may be less relevant to successful speech perception. Given the observations that some developmental language disorders, such as dyslexia, may be associated with impaired sensitivity to syllable stress^10–12^, future directions may examine this MMN (in)sensitivity in children and adults with dyslexia.

ERP responses to formal and temporal deviants both suggest that the probability of occurrence within a language has an impact on how we process speech, with more probable constructions being processed more readily. In the case of phonotactic probability, we observe this as more rapid change detection in the form of a shorter MMN peak latency. For syllable stress, we do not observe a statistically significant modulation of the MMN. The current ERP analysis focused specifically on the MMN, however future investigations may explore oscillatory patterns underlying these MMN modulations (see review on oscillatory mechanisms underlying predictions by Arnal & Giraud^4^). For instance, the observed MMN for temporal deviants may be the result of disrupted beta synchronization to the syllable stress. Additionally, examining gamma band modulations of formal deviant processing may shed light on differential processes underlying short vs. long-term predictions (established based on repeating standards preceding the deviant, and the formal structure of the language acquired during development, respectively).

It is worth noting that the relative frequencies for the “high” and “low” probability items in our manipulations differ for phonotactic probability and syllable stress. While the frequency ratio for HPP vs LPP items is 12.09, that for SylStr1 vs SylStr2 3.96 (Table 1). It is possible that the difference in frequency between more and less predictable items may influence the degree or morphology of the MMN response, which may in part explain why we observe different MMN patterns in response to formal and temporal deviants. However, it is difficult to directly compare these relative frequencies to each other, as they describe the occurrence of distinct linguistic features. Moreover, while many possible combinations of phoneme clusters exist, syllable stress in bisyllabic words remains binary: either the first, or the second syllable is stressed. Nevertheless, it would be interesting to investigate how the systematic variation of phonotactic probability may affect the MMN response, and how this compares to effects of syllable stress, as previous studies have also been limited to a global categorization of “high” vs. “low” probability^36–38^.

The lack of clear interaction between phonotactic probability and syllable stress, and the different response patterns for formal and temporal deviants (latency effect vs. no modulation of MMN) may indicate that these features are processed via independent, parallel mechanisms. This notion is supported by previous evidence from behavioural and neural findings. Processing of formal and temporal regularities in language appear to develop at different time scales. Infants are already sensitive to the rhythmic properties of their native language in the early days after birth^13^, while sensitivity to its phonological structure, including phonotactics, does not emerge until later in the first year (~6–8 months)^15–17,53^. In adults, both phonotactic probability and syllable stress have been shown to modulate performance on nonword repetition, with both high phonotactic probability and “typical” stress patterns improving performance^31^. These features are also associated with distinct neural correlates. For example, the processing of the formal and temporal structure of speech operate via separate oscillatory mechanisms, with neural oscillations in high frequency bands associated with phonological and syntactic encoding, and those in lower frequency bands with tracking the rhythmic structure^4,54^. These processes are further thought to engage distinct functional networks, where the formal structure of the signal is associated with the more classical auditory and speech processing pathways, and the temporal event structure is transmitted via the motor system, including cerebellar and supplementary motor areas^6,7^.

The paradigm developed here will further aim at studying the role of formal and temporal predictions in individuals with dyslexia who show reduced sensitivity to neural and behavioural measures of these features. Existing theories on the origin of dyslexia implicate deficits in the processing of formal^55–58^ or temporal^10,59^ structure of speech, or the combination of the two in the formation of cross-modal representations^60,61^. Testing our paradigm in children with normal and impaired reading development may help characterize differences in the processing of formal and temporal predictions that are critical to fluent reading skills. In addition to shedding light on the mechanisms and neural correlates of language and reading development, these investigations could be valuable in optimizing the training of pre-reading language skills in kindergarten and/or interventions to facilitate the acquisition of reading skills in children with dyslexia.

## Supplementary information

Supplementary Information.

## Data Availability

The datasets generated during and/or analysed during the current study are available from the corresponding author on reasonable request.
