# Supplementary material for: ERP mismatch response to phonological and temporal regularities in speech
Source: Sci Rep. 2020 Jun 18;10:9917. doi: 10.1038/s41598-020-66824-x (PMC7303198; doi:10.1038/s41598-020-66824-x)
Supplement: Supplementary file 1 — Supplementary Information. [file 41598_2020_66824_MOESM1_ESM.docx]

**Supplementary Materials to:**

**ERP Mismatch response to phonological and temporal regularities in speech**

Alexandra K. Emmendorfer*^,1,2,3^, Joao M. Correia^1,2,4^, Bernadette M. Jansma^1,2^, Sonja A. Kotz^3^, Milene Bonte^1,2^

1 Dept of Cognitive Neuroscience, Faculty of Psychology and Neuroscience, Maastricht University, Maastricht, The Netherlands

2 Maastricht Brain Imaging Center, Faculty of Psychology and Neuroscience, Maastricht University, Maastricht, The Netherlands

3 Dept of Neuropsychology and Psychopharmacology, Faculty of Psychology and Neuroscience, Maastricht University, Maastricht, The Netherlands

4 Centre for Biomedical Research (CBMR)/Department of Psychology, Universidade do Algarve, Portugal

*** Corresponding Author**:

Alexandra K. Emmendorfer, M.Sc.

Faculty of Psychology and Neuroscience, Maastricht University

Oxfordlaan 55, 6229 EV Maastricht, The Netherlands

E-mail: [a.emmendorfer@maastrichtuniversity.nl](mailto:a.emmendorfer@maastrichtuniversity.nl)

Tel: +31 433 88 21 59

Supplementary Information

Abbreviations:

FD = Formal Deviant

TD = Temporal Deviant

SD = Standard

MeanAmp = Mean Amplitude

HPP = high phonotactic probability

LPP = low phonotactic probability

SylS1 = first syllable stress

SylS2 = second syllable stress

FC = frontocentral ROI

CP = centroparietal ROI

Table S1. Formal deviants: Descriptive statistics of mean amplitude

| **PhonProb** | **SylStr** | **Cond** | **ROI** | **Mean** | **SD** | **N** |
| --- | --- | --- | --- | --- | --- | --- |
| HPP | SylS1 | FD | CP | -2.3060 | 1.50386 | 24 |
|  |  |  | FC | -2.5090 | 1.68411 | 24 |
|  |  | SD | CP | -.8236 | 1.55834 | 24 |
|  |  |  | FC | -1.0558 | 1.80899 | 24 |
|  | SylS2 | FD | CP | -1.5318 | 1.70586 | 24 |
|  |  |  | FC | -1.5195 | 1.96688 | 24 |
|  |  | SD | CP | -.2122 | 1.35312 | 24 |
|  |  |  | FC | -.2484 | 1.50607 | 24 |
| LPP | SylS1 | FD | CP | -2.0267 | 2.02642 | 24 |
|  |  |  | FC | -2.5083 | 2.54579 | 24 |
|  |  | SD | CP | -.9220 | 1.88350 | 24 |
|  |  |  | FC | -1.0999 | 2.20176 | 24 |
|  | SylS2 | FD | CP | -2.4064 | 1.45576 | 24 |
|  |  |  | FC | -2.8504 | 1.90247 | 24 |
|  |  | SD | CP | -.8729 | 1.13034 | 24 |
|  |  |  | FC | -.9649 | 1.32014 | 24 |

Table S2: Formal deviants: Descriptive statistics of peak latency

| **PhonProb** | **SylStr** | **Mean** | **SD** | **N** |
| --- | --- | --- | --- | --- |
| HPP | SylS1 | .198667 | .0536743 | 24 |
|  | SylS2 | .174500 | .0451750 | 24 |
| LPP | SylS1 | .202667 | .0495077 | 24 |
|  | SylS2 | .216000 | .0428384 | 24 |

Table S3. Formal deviants: Results of 2x2x2x2 repeated measures ANOVA on MMN amplitudes (PhonProb x SylStr x Cond x ROI). P-values adjusted with Bonferroni-Holm correction.

|  | F (1,23) | p (adj) |
| --- | --- | --- |
| PhonProb | 5.667 | 0.388 |
| SylStr | 3.217 | 0.814 |
| Cond | 107.642 | **< 0.001 ***** |
| ROI | 4.838 | 0.456 |
| PhonProb x SylStr | 3.510 | 0.814 |
| PhonProb x Cond | 0.187 | 1.000 |
| SylStr x Cond | 0.465 | 1.000 |
| PhonProb x SylStr x Cond | 1.430 | 0.814 |
| PhonProb x ROI | 3.106 | 0.814 |
| SylStr x ROI | 3.387 | 1.000 |
| PhonProb x SylStr x ROI | 0.487 | 1.000 |
| Cond x ROI | 1.897 | 1.000 |
| PhonProb x Cond x ROI | 8.568 | 0.112 |
| SylStr x Cond x ROI | 0.013 | 1.000 |
| PhonProb x SylStr x Cond x ROI | 0.061 | 1.000 |

Table S4: Formal deviants: Results of 2x2 repeated measures ANOVA on MMN peak latency (PhonProb x SylStr). P-values adjusted with Bonferroni-Holm correction.

|  | Peak Latency | |
| --- | --- | --- |
|  | F(1,23) | p (adj) |
| PhonProb | 16.249 | **0.0016 **** |
| SylStr | 0.190 | 0.667 |
| PhonProb x SylStr | 3.342 | 0.162 |

Table S5: Temporal deviants: Descriptive statistics of mean amplitude

| **PhonProb** | **SylStr** | **Cond** | **ROI** | **Mean** | **SD** | **N** |
| --- | --- | --- | --- | --- | --- | --- |
| HPP | SylS1 | TD | CP | -.8900 | 1.49488 | 24 |
|  |  |  | FC | -.6217 | 2.00712 | 24 |
|  |  | SD | CP | .6516 | 1.55185 | 24 |
|  |  |  | FC | .9468 | 1.93654 | 24 |
|  | SylS2 | TD | CP | -.5859 | 1.49901 | 24 |
|  |  |  | FC | -.6033 | 1.65122 | 24 |
|  |  | SD | CP | .3109 | 1.27811 | 24 |
|  |  |  | FC | .4742 | 1.66308 | 24 |
| LPP | SylS1 | TD | CP | -1.2490 | 1.49632 | 24 |
|  |  |  | FC | -1.1961 | 2.09801 | 24 |
|  |  | SD | CP | .2065 | 1.45071 | 24 |
|  |  |  | FC | .4951 | 1.79586 | 24 |
|  | SylS2 | TD | CP | -.9743 | 1.58432 | 24 |
|  |  |  | FC | -.8575 | 1.63777 | 24 |
|  |  | SD | CP | .2976 | 1.70135 | 24 |
|  |  |  | FC | .4545 | 1.82001 | 24 |

Table S6: Temporal deviants: Descriptive statistics of peak latency

| **PhonProb** | **SylStr** | **Mean** | **SD** | **N** |
| --- | --- | --- | --- | --- |
| HPP | SylS1 | .25967 | .046482 | 24 |
|  | SylS2 | .25533 | .069869 | 24 |
| LPP | SylS1 | .26400 | .058562 | 24 |
|  | SylS2 | .28850 | .057536 | 24 |

Table S7. Temporal deviants: Results of 2x2x2x2 repeated measures ANOVA on MMN amplitudes (PhonProb x SylStr x Cond x ROI). P-values adjusted with Bonferroni-Holm correction.

|  | F (1,23) | P (adj) |
| --- | --- | --- |
| PhonProb | 1.719 | 1.000 |
| SylStr | 0.013 | 1.000 |
| Cond | 78.159 | **<0.001 ***** |
| ROI | 3.573 | 0.923 |
| PhonProb x SylStr | 0.841 | 1.000 |
| PhonProb x Cond | 0.981 | 1.000 |
| SylStr x Cond | 5.360 | 0.420 |
| PhonProb x SylStr x Cond | 0.332 | 1.000 |
| PhonProb x ROI | 0.048 | 1.000 |
| SylStr x ROI | 1.811 | 1.000 |
| PhonProb x SylStr x ROI | 1.069 | 1.000 |
| Cond x ROI | 2.000 | 1.000 |
| PhonProb x Cond x ROI | 0.095 | 1.000 |
| SylStr x Cond x ROI | 0.020 | 1.000 |
| PhonProb x SylStr x Cond x ROI | 1.258 | 1.000 |

Table S8: Results of 2x2 repeated measures ANOVA on MMN peak latency (PhonProb x SylStr). P-values adjusted with Bonferroni-Holm correction.

|  | Peak Latency | |
| --- | --- | --- |
|  | F(1,23) | p (adj) |
| PhonProb | 2.039 | 0.501 |
| SylStr | 0.895 | 0.501 |
| PhonProb x SylStr | 1.467 | 0.501 |
